# A pectin/chitosan/zinc oxide nanocomposite for adsorption/photocatalytic remediation of carbamazepine in water samples†

**DOI:** 10.1039/d0ra08010a

**Published:** 2020-11-09

**Authors:** Olivia A. Attallah, Mahmoud Rabee

**Affiliations:** Pharmaceutical Chemistry Department, Faculty of Pharmacy, Heliopolis University Cairo – Belbeis Desert Road, El Salam Cairo 11777 Egypt olivia.adly@hu.edu.eg

## Abstract

The present study investigates a synergistic adsorption/photodegradation technique catalyzed by a pectin/chitosan/zinc oxide (Pec/CS/ZnO) nanocomposite for the removal of carbamazepine (CBZ) in aqueous solutions under direct sunlight. The Pec/CS/ZnO nanocomposite was prepared by an inotropic gelation method and was characterized using different techniques. The adsorption/photocatalytic activity of the Pec/CS/ZnO nanocomposite for the remediation of CBZ was optimized using Box–Behnken design under response surface methodology. The examined parameters included the amount of Pec/CS/ZnO nanocomposite (0.25–0.75 g L^−1^), pH (4–10), and run time for adsorption/photo-irradiation (1–5 hours). The efficiency of CBZ degradation was calculated in terms of changes in CBZ concentration using a validated chromatographic assay. The optimum conditions for the remediation of CBZ were 0.5 g L^−1^ Pec/CS/ZnO nanocomposite, pH 4, and 3 hour run time. Under such conditions, the degradation efficiency of 10 mg L^−1^ CBZ was found to be 69.5% with a rate constant (*k*) of 0.00737 min^−1^ and half-life time of 94 min. The efficiency of the Pec/CS/ZnO nanocomposite for CBZ remediation was found to be stable and consistent after three cycles of reuse. The presence of other pharmaceutical contaminants such as acetaminophen in wastewater samples was also investigated. The efficiency of CBZ degradation was not significantly affected by the addition of acetaminophen in a 0–15 mg L^−1^ concentration range which confirmed the selectivity and efficiency of the proposed method for CBZ degradation and removal.

## Abstract 2

The present study investigates a synergistic adsorption/photodegradation technique catalyzed by a pectin/chitosan/zinc oxide (Pec/CS/ZnO) nanocomposite for the removal of carbamazepine (CBZ) in aqueous solutions under direct sunlight.

## Graphical abstract

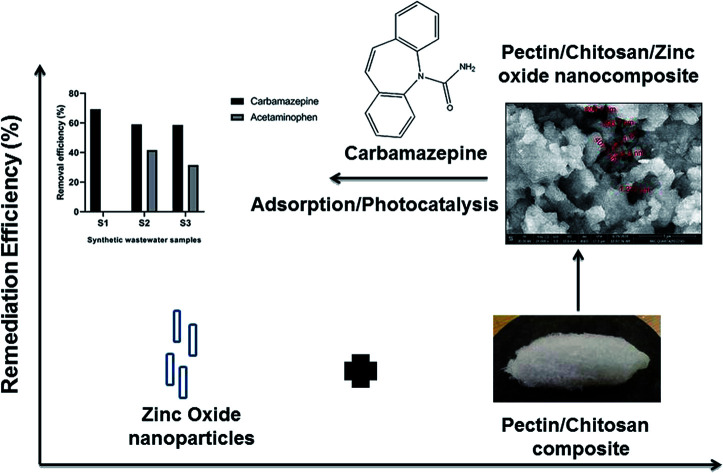

## 1. Introduction

Pharmaceuticals have long saved the lives of millions and lengthened their life spans. Through the years, pharmaceuticals have been able to cure deadly diseases, prevent the occurrence of certain illnesses and improve quality of life.^1^ Nevertheless, despite their success and importance, pharmaceuticals have emerged as rapidly growing environmental contaminants.^2^ Recently, residues of pharmaceutical contaminants have been found in most environmental matrices and they are considered as “compounds of emerging concern”.^1^ Pharmaceutical residues have very great potential to harm ecosystems and threaten human health.^2^ Consequently, different analytical techniques, such as UPLC/MS, LC-MS/MS, and GC-MS/MS have been developed to monitor the concentration of these residues in different environmental matrices.^1^ In addition, wastewater treatment plants (WWTPs) were designed to assist in their remediation process but their efficiency was less than 10% for certain pharmaceuticals.^3^

**Fig. 1:**
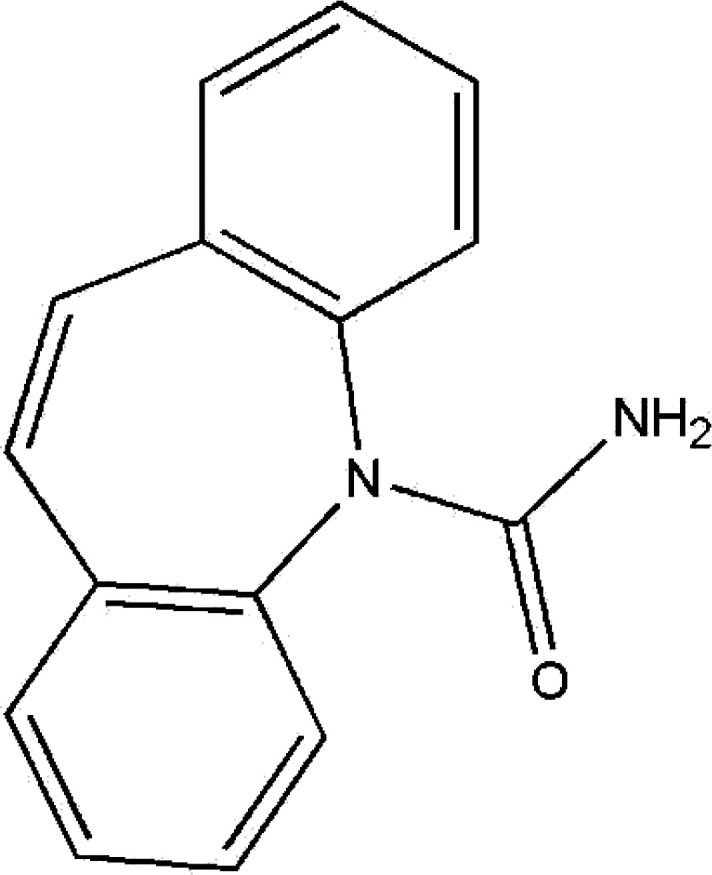
Chemical structure of carbamazepine (CBZ).

Carbamazepine (CBZ) (Fig. 1) is an anticonvulsant drug and is one of these pharmaceuticals described with very poor remediated efficiency by WWTPs.^1,2^ CBZ is a tricyclic neutral compound of lipophilic nature and is utilized in the treatment of trigeminal neuralgia, partial epilepsy and as an adjunct therapy in psychosis.^4^ Various routes of treatment have been proposed to overcome the inefficiency of WWTP in the removal of CBZ. Such approaches include advanced oxidation,^5–10^ membrane filtration,^11–13^ and ozonation.^14–17^

To date, photocatalytic degradation has proven to efficiently degrade several pharmaceuticals from natural-waters and waste-waters.^18^ The process of photocatalysis usually occurs in the presence of a catalyst such as zinc oxide (ZnO) and titanium dioxide (TiO_2_) nanoparticles which have been thoroughly tested and showed very promising results.^5,18–25^

Moreover, it was recently suggested that to treat organic pollutants in waste-waters and natural-waters, multifunctional, advanced materials are required.^26^ In this sense, hybrid organic/inorganic nanocomposites are of great interest for environmental remediation. Such materials possess multi functionality due to the incorporation of various compounds in their matrices.^27^ Accordingly, these nanocomposites can serve in combined adsorption/photocatalysis processes. The organic portion of the nanocomposite incorporates polymers like chitosan, polyaniline, cellulose, pectin, polyacrylamide and polystyrene.^26^ These polymers act as adsorbents, increase the exposed surface area for remediation and improve the mechanical properties of the inorganic portion of the composite. On the other hand, the inorganic counterpart such as ZnO and TiO_2_ is responsible for the composite photocatalytic activity.^26^

Several studies have evaluated the efficiency of organic/inorganic nanocomposites for the remediation of different contaminants in aqueous solutions. For instance, Gupta *et al.* fabricated a nanocomposite of pectin Zr(iv) seleno-tungsto-phosphate for synergistic adsorption/photocatalytic remediation of methylene blue and malachite green dyes from aqueous solution.^26^ They were able to reach a photocatalytic degradation percentage of 89.21% for methylene blue and 79.27% for malachite green after 3 hours of synergistic adsorption/photo-irradiation processes.^26^ Another study evaluated the efficiency of g-C_3_N_4_/Ag_2_CO_3_ hybrid photocatalyst for the remediation of methyl orange dye and found that the hybrid material showed enhanced photocatalytic activity than pure g-C_3_N_4_ and Ag_2_CO_3_.^28^ Fu *et al.* also demonstrated that the activity of a hybrid photocatalyst of C_3_N_4_/CdS is 20 times higher than those of individual C_3_N_4_ and CdS for the degradation of methyl orange.^29^ Yan *et al.* studied the activity of g-C_3_N_4_–TaON photocatalyst for the photodegradation of rhodamine B and found that the new hybrid photocatalyst has higher catalytic activity than the single-phase g-C_3_N_4_ or TaON.^30^

The main objective of this work is to study a synergetic adsorption/photocatalysis technique in the presence of an organic/inorganic nanocomposite for the remediation of CBZ as model pharmaceutical contaminant. Based on surveyed literature, the photo-degradation of CBZ is well documented and is a subject of massive research. Photocatalysis is considered one of the efficient techniques for the removal of CBZ and the presence of nanoparticles as TiO_2_ and ZnO enhances the photocatalytic process and shortens the time required for degradation.^18–20,22,23^

Here we focus on the synthesis of a novel organic/inorganic; pectin/chitosan/ZnO (Pec/CS/ZnO) nanocomposite having chitosan and pectin polymers acting as adsorbents for CBZ while inorganic nano ZnO is responsible for the photocatalytic degradation of CBZ. The incorporation of nano ZnO into pectin and chitosan to form nanocomposite is expected to impart unique functionalities to the prepared nanocomposite and improve its efficiency in CBZ remediation. The effect of selected parameters; amount of Pec/CS/ZnO nanocomposite, pH and adsorption/photo-irradiation time (run time) on the efficiency of CBZ degradation has been studied and optimized using Box–Behnken design. The efficacy of presence of other contaminants in wastewaters on the proposed remediation technique was also evaluated and acetaminophen was selected as model contaminant.

To the best of our knowledge, there are no studies that discuss the development and optimization of a synergistic adsorption/photocatalytic technique using Pec/CS/ZnO nanocomposite for the efficient remediation of organic pharmaceutical contaminants as CBZ.

## 2. Experimental

### 2.1. Materials

Zinc sulphate heptahydrate (ZnSO_4_·7H_2_O) and pectin from the rind of citrus or apple (galacturonic acid ≥ 74.0%) were obtained from Fisher Scientific (USA). Chitosan of low molecular weight (89.9% degree of dealkylation) was purchased from (Primex ehf, Chitoclear, Iceland), calcium chloride dihydrate (CaCl_2_·2H_2_O) was obtained from Sigma Aldrich Co. (USA). Pharmaceutical grade carbamazepine (CBZ) and acetaminophen were supplied by Novartis Pharmaceuticals (Cairo, Egypt) and certified to have 99.7% purity. HPLC grade acetonitrile was purchased from Honeywell (Germany) and HPLC grade water was obtained from LiChrosolv (Germany).

### 2.2. Preparation of Pec/CS/ZnO nanocomposite

#### 2.2.1. Preparation of ZnO nanoparticles

ZnO nanoparticles were prepared using a direct precipitation method proposed by Ghorbani *et al.* with slight modification.^31^ Briefly, aqueous solution (0.2 M) of zinc sulphate (ZnSO_4_·7H_2_O) and (0.4 M) solution of sodium hydroxide (NaOH) were prepared in distilled water. The NaOH solution was slowly added into zinc sulphate solution under vigorous stirring at room temperature until pH reached 9. The mixture was kept under stirring for 2 hours and the white precipitate was then separated *via* centrifugation at 5000 rpm for 15 min and washed three times with distilled water. The obtained product was then calcined at 250 °C for 3 hours.

#### 2.2.2. Preparation of Pec/CS/ZnO nanocomposite

Inotropic gelation technique was used to prepare Pec/CS/ZnO nanocomposite at room temperature.^32^ To 60 mL Pec solution (0.44% (w/v)), 0.5 g of the prepared ZnO nanoparticles was added and left under magnetic stirring for 30 min to ensure homogenous dispersion. CS solution (2% (w/v)) was prepared in 1% acetic acid (w/v), while calcium chloride (CaCl_2_) solution (0.5% (w/v)) was prepared in distilled water. 15 mL of (0.28% (w/v)) CS and (0.064% (w/v)) CaCl_2_ mixture solution was slowly added to the 60 mL Pec/ZnO dispersion. The pH was adjusted at 5.5 and the mixture solution was left under stirring for 60 min. The prepared Pec/CS/ZnO nanocomposite was then isolated *via* centrifugation at 5000 rpm for 20 min, followed by washing several times in distilled water. Finally, Pec/CS/ZnO nanocomposite was dispersed in distilled water and dried at 50 °C for 72 hours.

### 2.3. Adsorption/photocatalytic experiments of CBZ

The adsorption/photocatalytic experiments were carried out on CBZ as model pharmaceutical contaminant in aqueous samples using batch process. The initial concentration of CBZ was kept at 10 mg L^−1^. Determined amounts of Pec/CS/ZnO nanocomposite were added to the test samples and pH was adjusted using 0.1 M hydrochloric acid and sodium hydroxide solutions. Samples were then placed in a glass chamber jacketed with thermostat water circulation to maintain constant temperature at 25 °C and were exposed directly to sunlight to allow a synergistic adsorption/photocatalysis process to occur. After the specified times samples were removed, filtered and the concentration of CBZ in the supernatant solutions was determined *via* a validated chromatographic assay.

The efficiency of degradation (%) was calculated using eqn (1).^33^1
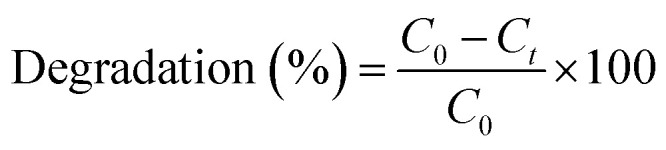


The rate of photocatalytic degradation of CBZ was then tested against pseudo first order kinetic model using eqn (2):^34^2
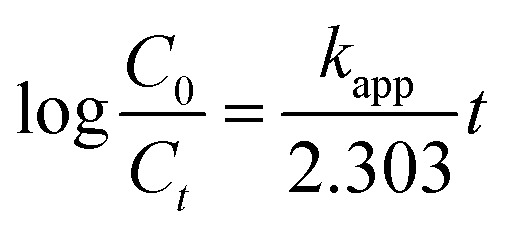
where *k*_app_ is the apparent rate constant, *C*_0_ and *C*_*t*_ are the concentrations of CBZ before treatment and at time *t*, respectively and the half life time as 
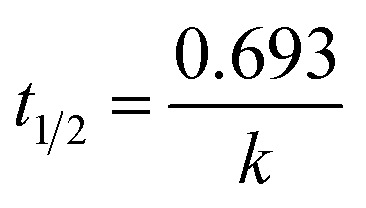
.^34^

### 2.4. Chromatographic analysis

Stock standard solutions of both CBZ and acetaminophen (0.1 mg mL^−1^) were prepared in 100 mL methanol. Aliquots of stock solutions were withdrawn and diluted with distilled water to prepare working standard solutions of 1 to 100 μg mL^−1^ concentration range. HPLC chromatographic separation was achieved using a HiQsil C18 HS column (250 × 4.6 mm, 5 mm) on HPLC system model 1100 (Agilent Technologies, USA) with variable wavelength detector and an auto sampler. Analyses were performed using isocratic elution with a mobile phase of water : acetonitrile (60% : 40% v/v, 1.5 mL min^−1^) at ambient temperature and 20 μL injection volume. Detection was carried out at 284 nm for CBZ and 240 nm for acetaminophen using variable wavelength detector. Calibration curves were plotted over concentration range of 1–25 μg mL^−1^ for CBZ and acetaminophen and validation parameters were calculated according to ICH-guidelines.

### 2.5. Design of experiment

Three-factor, three-level Box–Behnken design (BBD) (Design Expert, trial version 10.0.5.0, Stat-Ease Inc., Minneapolis, MN) was implemented for the optimization of CBZ remediation using Pec/CS/ZnO nanocomposite. The design involved fifteen experimental runs with three replicated center points. The chosen independent factors were the amount of Pec/CS/ZnO nanocomposite (*X*_1_), pH (*X*_2_), and run time (*X*_3_) and were varied at three levels. The response or dependent variable studied was taken as the efficiency of CBZ degradation (%). The independent factors and ranges for each independent factor were based on available literature and preliminary experiments. Nominated ranges are shown in (Table 1). Other parameters like temperature and agitation speed were kept constant in all experiments.

**Table:** Variables and levels in Box–Behnken experimental design for CBZ degradation and removal

Independent variables	Level		
	−1	0	1
*X*_1_: amount of Pec/CS/ZnO (g L^−1^)	0.25	0.50	0.75
*X*_2_: pH	4	7	10
*X*_3_: run time (hour)	1	3	5

### 2.6. Reusability of Pec/CS/ZnO nanocomposite

To test the degradation stability and reusability of Pec/CS/ZnO nanocomposite, the adsorption/photocatalytic degradation experiments were carried out in triplicate under the same conditions (0.5 g L^−1^ Pec/CS/ZnO nanocomposite, pH 4 and 3 hour run time) on 10 mg L^−1^ CPZ samples. After each use, Pec/CS/ZnO nanocomposite were placed in a beaker of 50 mL 5% (v/v) methanol/HCl for one hour to remove any adsorbed CBZ. The solvent was then decanted and Pec/CS/ZnO nanocomposite was dried in an oven at 60 °C for 2 hours. The nanocomposite was reused for two more times for CBZ adsorption/photodegradation, in the aforementioned conditions.

### 2.7. Application on synthetic wastewater samples

The efficiency of the proposed synergistic adsorption/photocatalytic method for the remediation of CBZ in aqueous solutions in the presence of other contaminants was evaluated. Synthetic wastewater samples were prepared in distilled water and adsorption/photocatalytic experiments were performed using the optimized conditions obtained from BBD. The synthetic wastewater samples were spiked with CBZ and acetaminophen as model pharmaceutical contaminants in the wastewater samples. Table 2 shows the composition of the synthetic wastewater samples. Pec/CS/ZnO nanocomposite (0.5 g L^−1^) was added to the synthetic wastewater samples and pH was adjusted at 4. Samples were then added to the glass chamber where temperature was kept constant at 25 °C. The synergistic adsorption/photocatalytic process was performed for 3 hours in direct sunlight. Samples were then collected, centrifuged and the concentrations of CBZ and acetaminophen left in the supernatant solutions of each sample were determined by the validated chromatographic assay.

**Table:** Composition of synthetic wastewater samples

Samples (S)	CBZ concentration (mg L^−1^)	Acetaminophen concentration (mg L^−1^)
S1	10	0
S2	10	10
S3	10	15

## 3. Results and discussion

### 3.1. Characterization of Pec/CS/ZnO nanocomposite

**Fig. 2:**
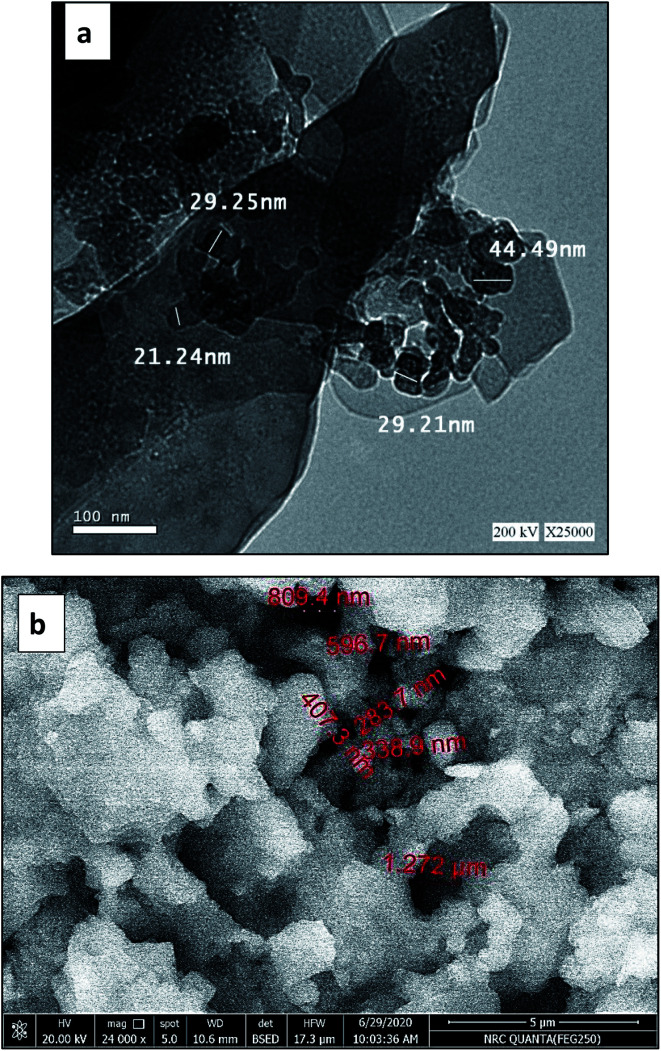
(a) TEM image of ZnO nanoparticles, (b) SEM image of Pec/CS/ZnO nanocomposite.

Transmission electron microscopy (TEM) on Tecani G20, FEI transmission electron microscope (USA) and scanning electron microscopy (SEM) on a Zeiss Instrument (Germany) were used for the morphological study of Pec/CS/ZnO nanocomposite. Fig. 2(a) shows the TEM image of ZnO nanoparticles prepared by precipitation method. In this figure, the particles showed a formation of clusters having an average diameter of 30 nm. Fig. 2(b) shows the SEM image of Pec/CS/ZnO nanocomposite consisting of particles which are arranged as porous, granular-like structure. The particle size of Pec/CS/ZnO nanocomposite was in the range of 300–800 nm in diameter.

**Fig. 3:**
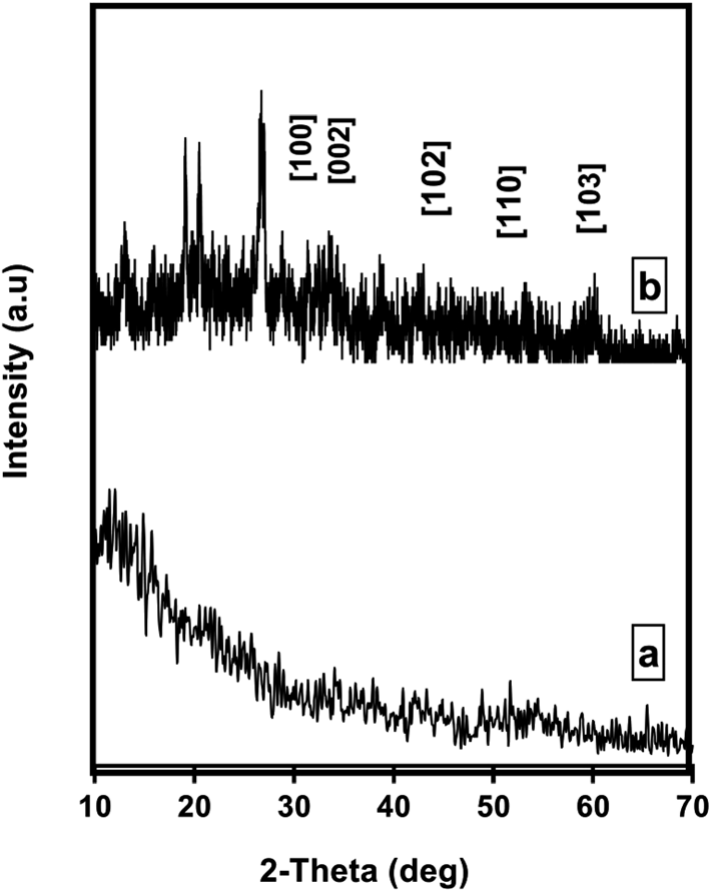
XRD pattern of (a) Pec/CS and (b) Pec/CS/ZnO nanocomposites.

Crystallographic properties of Pec/CS and Pec/CS/JO nanocomposites were evaluated using X-ray diffraction (XRD) over a 2*θ* range from 10 to 70° (2*θ*) on a Bruker AXS D8 (Germany). As shown in Fig. 3(a), XRD pattern of Pec/CS nanocomposite showed a broad typical hump of amorphous material indicating the noncrystalline nature of the nanocomposite. The XRD pattern of Pec/CS/ZnO nanocomposite demonstrated the presence of diffraction peaks that were indexed to those of hexagonal ZnO (JCPDS card, no. 80-0075). Noticeably the peaks were somehow broadened which suggested that Pec/CS/ZnO nanocomposite was also amorphous in nature proving the interaction between ZnO NPs and Pec/CS composite.

**Fig. 4:**
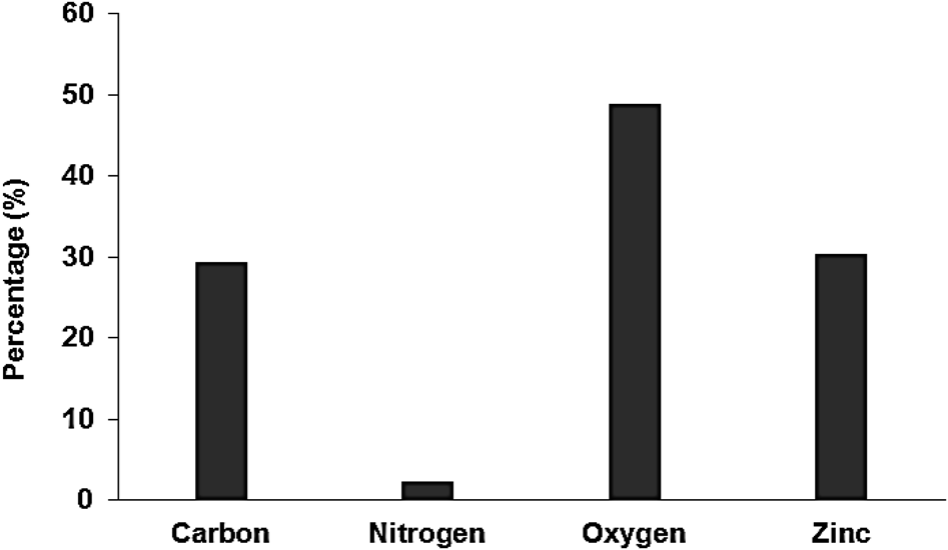
EDX analysis of Pec/CS/ZnO nanocomposite.

Analysis of the chemical composition of Pec/CS/ZnO nanocomposite was implemented by Energy Dispersion X-ray technique (EDX) in a Zeiss Instrument (Germany). Fig. 4 reveals the presence of C, O, N and Zn elements in Pec/CS/ZnO nanocomposite with a 30% Zn element proportion in the tested samples. Such results indicate the incorporation and significant dispersion of ZnO NPs within Pec/CS composite matrix.

**Fig. 5:**
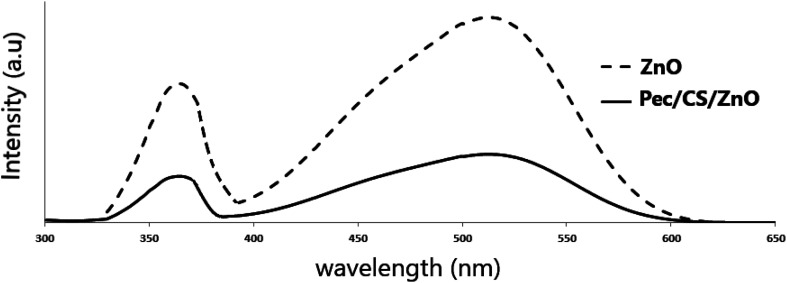
Photoluminescence spectra of ZnO NPs and Pec/CS/ZnO nanocomposite.

The photoluminescence spectra of ZnO NPs and Pec/CS/ZnO nanocomposites obtained at the excitation wavelength of 325 nm at room temperature using PerkinElmer LS 55 Luminescence spectrometer are demonstrated in Fig. 5. The emission peak at 365 nm is ascribed for electron transition between shallow donor level of Zn interstitial to top level of the valence band.^35^ The emission band at 515 nm is due to the recombination of photo generated holes by electrons in singly occupied oxygen vacancies.^35^ Noticeably, the luminescence properties of Pec/CS/ZnO nanocomposite have been suppressed compared to pure ZnO which could be due to the transition effect of higher energy and charge between Pec/CS and ZnO.^36^ The band gap energy of Pec/CS/ZnO nanocomposite was also slightly shifted which could be attributed to the interaction between Pec/CS and ZnO in the nanocomposite.^36^

**Fig. 6:**
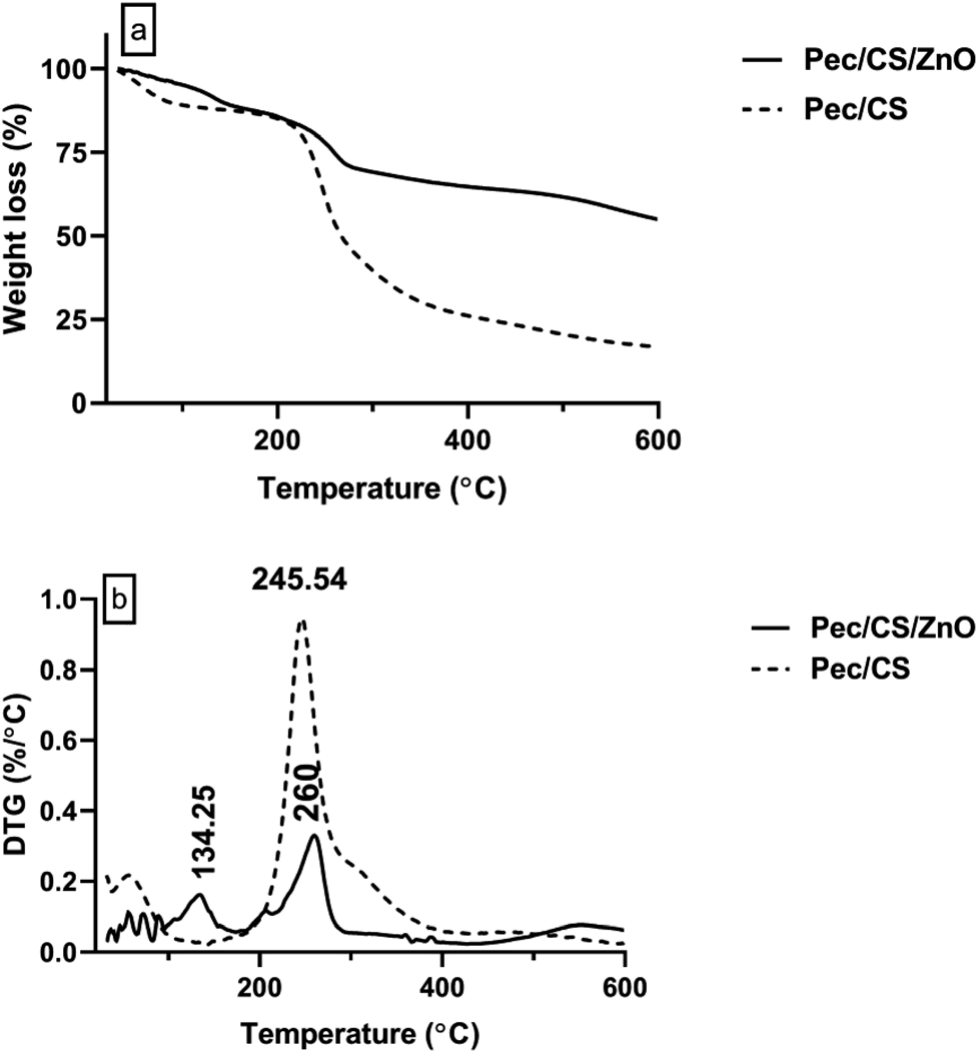
(a) TGA and (b) DTG of Pec/CS and Pec/CS/ZnO nanocomposite.

Thermogravimetric analysis was performed on a Q50 Thermogravimetric Analyzers, TA, USA, in the temperature range 20–600 °C under N_2_ atmosphere. Fig. 6 shows the results of thermogravimetric analysis (TGA and DTG) of Pec/CS and Pec/CS/ZnO nanocomposites. A clear difference can be observed between the two composites thermograms indicating the successful incorporation of ZnO nanoparticles within Pec/CS composite matrix. In the DTG curve of Pec/CS/ZnO nanocomposite, an initial peak at 134.25 °C related to moisture evaporation can be observed with up to 20% weight loss. A second strong peak starting at 220 °C reaching its maximum at 260 °C was also found on the DTG curve. The appearance of this peak is attributed to the thermal de-polymerization of Pec and CS chains in the nanocomposite. Noticeably, there was a 15 °C temperature difference for the appearance of this depolymerization peak between Pec/CS/ZnO nanocomposite and Pec/CS composite. Such results indicated that the de-polymerization process was hindered to some degree due to the presence of strong interactions between Pec/CS and ZnO molecules.^27^

### 3.2. Chromatographic analysis

**Fig. 7:**
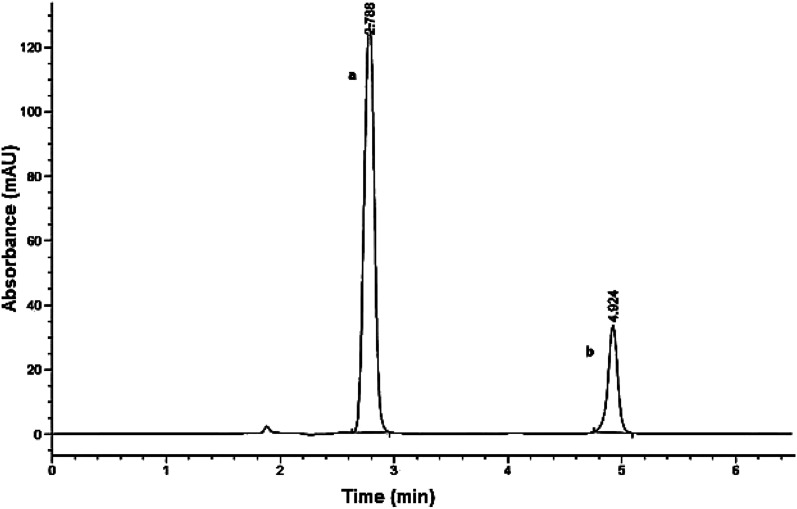
HPLC chromatogram of (a) acetaminophen at retention time = 2.7 min and (b) carbamazepine (CBZ) at retention time = 4.9 min.

Different chromatographic conditions were studied to get the optimum separation pattern of CBZ and acetaminophen. Isocratic elution with a mobile phase of water : acetonitrile 60% : 40% v/v was found to be the most efficient for separation purposes. The run time was 6.5 min, with flow rate of 1.5 mL min^−1^. As shown in Fig. 7, the retention times were found to be 2.7 and 4.9 min for acetaminophen and CBZ, respectively. System suitability parameters were also calculated as demonstrated in Table 3 and validation parameters performed per ICH guidelines are summarized in Table 4. The regression equations obtained from the chromatographic assay were used to calculate the concentrations of CBZ and acetaminophen throughout the whole study.

**Table:** System suitability tests for chromatographic method for the simultaneous determination of CBZ and acetaminophen in their mixture

Parameters	Obtained value		
	Carbamazepine	Acetaminophen	Reference value
Retention time (*t*_R_)	4.924	2.788	
Symmetry factor (*A*_s_)	1	1.1	*T* ≤ 2
Theoretical number plates (*N*)	50 094	12 288	*N* ≥ 2000
Capacity factor (*K*)	3.476	1.534	1–10 acceptable
Resolution (*R*_s_)	22.65		*R*_s_ ≥ 1.5
Selectivity factor (*α*)	2.26		*α* > 1

**Table:** Chromatographic method validation for the determination of laboratory prepared standards of CBZ and acetaminophen

Item	CBZ	Acetaminophen
Retention time	4.92 min	2.78 min
Wavelength of detection	284 nm	240 nm
Range of linearity	2–25 μg mL^−1^	2–25 μg mL^−1^
Regression equation	*A* = 41.5*C* + 2.0991	*A* = 52.899*C* + 1.9322
Regression coefficient (*r*^2^)	0.9997	0.9996
LOD (μg mL^−1^)	0.593	0.533
LOQ (μg mL^−1^)	1.797	1.615
Accuracy mean ± SD	99.66 ± 0.54	100.84 ± 0.7045

**Precision**		
Intraday % RSD (*n* = 9)	0.33–0.77	0.08–0.29
Interday % RSD (*n* = 9)	0.28–0.70	0.05–0.46

### 3.3. Adsorption/photocatalytic experiments of CBZ

#### 3.3.1. Experimental design results

The efficiency of CBZ degradation was analyzed using response surface methodology to evaluate the effects of the studied variables and determine the optimized operating conditions for the remediation process. An experimental design plan was developed for the selected variables and the experimental runs constituted the variation of these variables based on the proposed design. The efficiency of CBZ degradation (%) after each run was determined and presented as response for the run as summarized in Table 5. Different regression models were fitted to the response data to test for the best fitting model and evaluate the significance of the effect of the studied variables.

**Table:** Experimental matrix and observed response for CBZ in BBD

Run	Independent variable			Dependent variable
	*X*_1_ (g L^−1^)	*X*_2_	*X*_3_ (hour)	*Y* (%)
1	0.5	7	3	67.88
2	0.25	7	5	33
3	0.5	10	5	66.80
4	0.75	7	5	59.10
5	0.5	7	3	68.00
6	0.5	7	3	68.50
7	0.5	4	5	58.18
8	0.25	4	3	52.00
9	0.75	10	3	53.50
10	0.5	10	1	47.18
11	0.75	4	3	55.50
12	0.25	7	1	46.50
13	0.5	4	1	62.20
14	0.75	7	1	30.88
15	0.25	10	3	47.30

Table 6 demonstrates the relation between the efficiency of CBZ degradation (*Y*) and the studied variables; amount of Pec/CS/ZnO nanocomposite (*X*_1_), pH (*X*_2_) and run time (*X*_3_). Based on the obtained results, the chosen model to explain CBZ remediation process was the quadratic model. The coefficients of the quadratic equation indicated that the amount of Pec/CS/ZnO nanocomposite and the run time together with their interactions had positive effects on the efficiency of CBZ degradation. The increase in pH value showed a negative effect on the remediation process while its interactions with both the amount of Pec/CS/ZnO nanocomposite and the run time had positive coefficient values. Such results indicated that the amount of Pec/CS/ZnO nanocomposite and the run time had more significant effects on CBZ degradation than that of pH. Consequently, it can be concluded that the amount of Pec/CS/ZnO nanocomposite and the run time are the most effective variables in CBZ remediation followed by pH.

**Table:** Statistical analysis of measured responses for CBZ degradation and removal

Fitting model	Factors	Coefficient	*p*-Value	ANOVA
Degradation efficiency (*Y*)	Intercept	68.13		*F* = 2765.5, *R*^2^ = 0.9994, model *p*-value < 0.0001, *p*-value of lack of fit = 0.6874
	*X*_1_	2.52	<0.0001	
	*X*_2_	−1.64	<0.0001	
	*X*_3_	3.79	<0.0001	
	*X*_1_ *X*_2_	0.68	0.0050	
	*X*_1_ *X*_3_	10.43	<0.0001	
	*X*_2_ *X*_3_	5.91	<0.0001	
	*X*_1_^2^	−16.14	<0.0001	
	*X*_2_^2^	0.085	0.5910	
	*X*_3_^2^	−9.62	<0.0001	

A sequential test was performed to test the adequacy of the quadratic model to describe CBZ remediation process. A high *F*-value of 2765.5 was obtained for the quadratic model indicating the effectiveness of the proposed model to explain the studied response. High coefficient of determination (*R*^2^ = 0.9998) was also obtained for the CBZ remediation process and the adjusted *R*^2^ value (0.9996) was in close agreement with the predicted one (*R*^2^ = 0.9983).

To determine the significance of the quadratic model at a confidence interval of 95%, analysis of variance (ANOVA) was performed. As shown in Table 6, the obtained probability value (*p*-value) of the selected model was less than 0.05 (*p*-value < 0.0001) indicating that the studied response was in good fit with the quadratic model. In addition, the lack of fit of the studied response was non-significant having a *p*-value greater than 0.1 (*p*-value = 0.6874). Such result is highly desirable as it conclude the suitability of the chosen model to discuss the studied response.

Moreover, the experimental values for the efficiency of CBZ degradation were compared with the predicted values proposed by the quadratic model to evaluate the correlation between them. As shown in Fig. S1.† The plots of the predicted and experimental values were more than 95% coherent. Such coherence indicates the good correlation between the experimental and predicted values and the ability of the studied variables to provide a good prediction for the CBZ remediation process.

#### 3.3.2. Response surface analysis

**Fig. 8:**
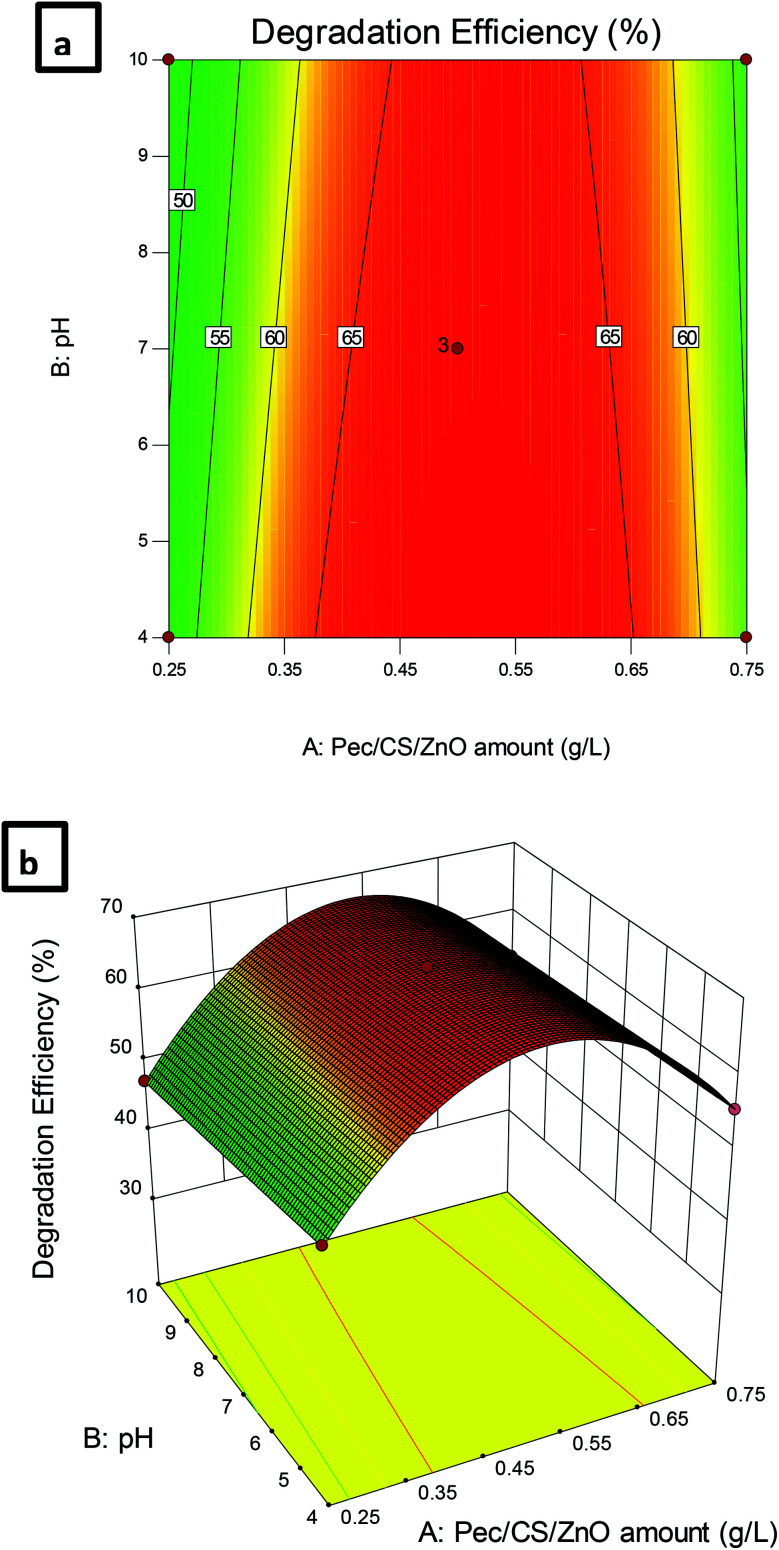
(a) Contour plot and (b) 3D plot showing the effect of Pec/CS/ZnO amount and pH on CBZ degradation efficiency.

Response surface analysis was performed and presented using contour and 3-D plots to estimate the effects of combinations of the studied variables on the efficiency of CBZ degradation. The dependency of CBZ degradation on pH and the amount of Pec/CS/ZnO nanocomposite is illustrated in Fig. 8. The effect of pH was consistent at high and low levels of Pec/CS/ZnO nanocomposite where the maximum percentage of CBZ degradation was observed at pH 4. Such result indicates that CBZ remediation process is favored at low pHs where the adsorption of CBZ (p*K*_a_ = 13.9) on Pec/CS/ZnO composite (point of zero charge 2.5 (data not shown)) is maximal and suggested to occur through charge forces in aqueous solution. Thus, with the increase of CBZ adsorption on Pec/CS/ZnO nanocomposite, the process of CBZ photo-degradation was more facilitated.^18^ The amount of Pec/CS/ZnO nanocomposite also had a significant effect on the remediation process at pH 4. An increase in the nanocomposite amount resulted in an increase in CBZ degradation reaching its maximum at 0.5 g L^−1^ nanocomposite and then CBZ degradation started to decrease with further increase in the nanocomposite amount. Such behavior could be attributed to the rapid agglomeration of the nanocomposite at elevated concentrations which led to a reduced ability of effective CBZ adsorption on the nanocomposite surface. Thus the rate of degradation reaction was decreased due to the decrease in the production of positive electron–hole and oxidizer radicals.^37^ Another explanation for such decrease in the efficiency of CBZ degradation could be due to the fact that very high concentrations of Pec/CS/ZnO nanocomposite produced large amounts of hydroxyl radicals causing more degradation of CBZ. The increase in CBZ degradation led to the production of large amounts of by-products that deposited on the surface of the nanocomposite resulting in an accelerated deactivation of the photocatalytic activity.^38^

**Fig. 9:**
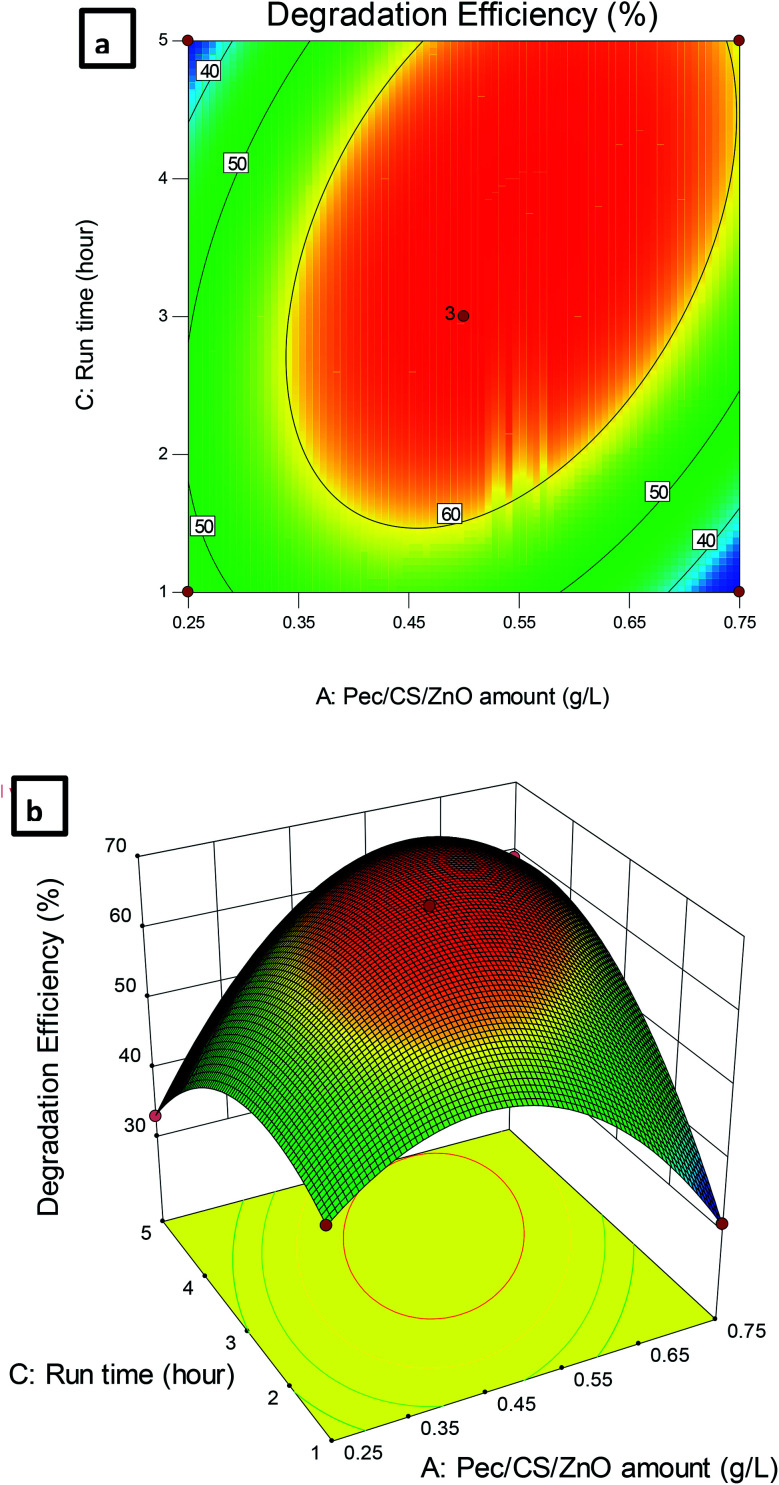
(a) Contour plot and (b) 3D plot showing the effect of Pec/CS/ZnO amount and run time on CBZ degradation efficiency.

The dependence of CBZ remediation on the amount of Pec/CS/ZnO nanocomposite and run time is shown in Fig. 9. The highest percentage of CBZ degradation was observed at 0.5 g L^−1^ nanocomposite and 3 hour run time. Further increase in the levels of both variables did not significantly improve the CBZ remediation process which indicated that an equilibrium situation was attained after 3 hours of treatment.

**Fig. 10:**
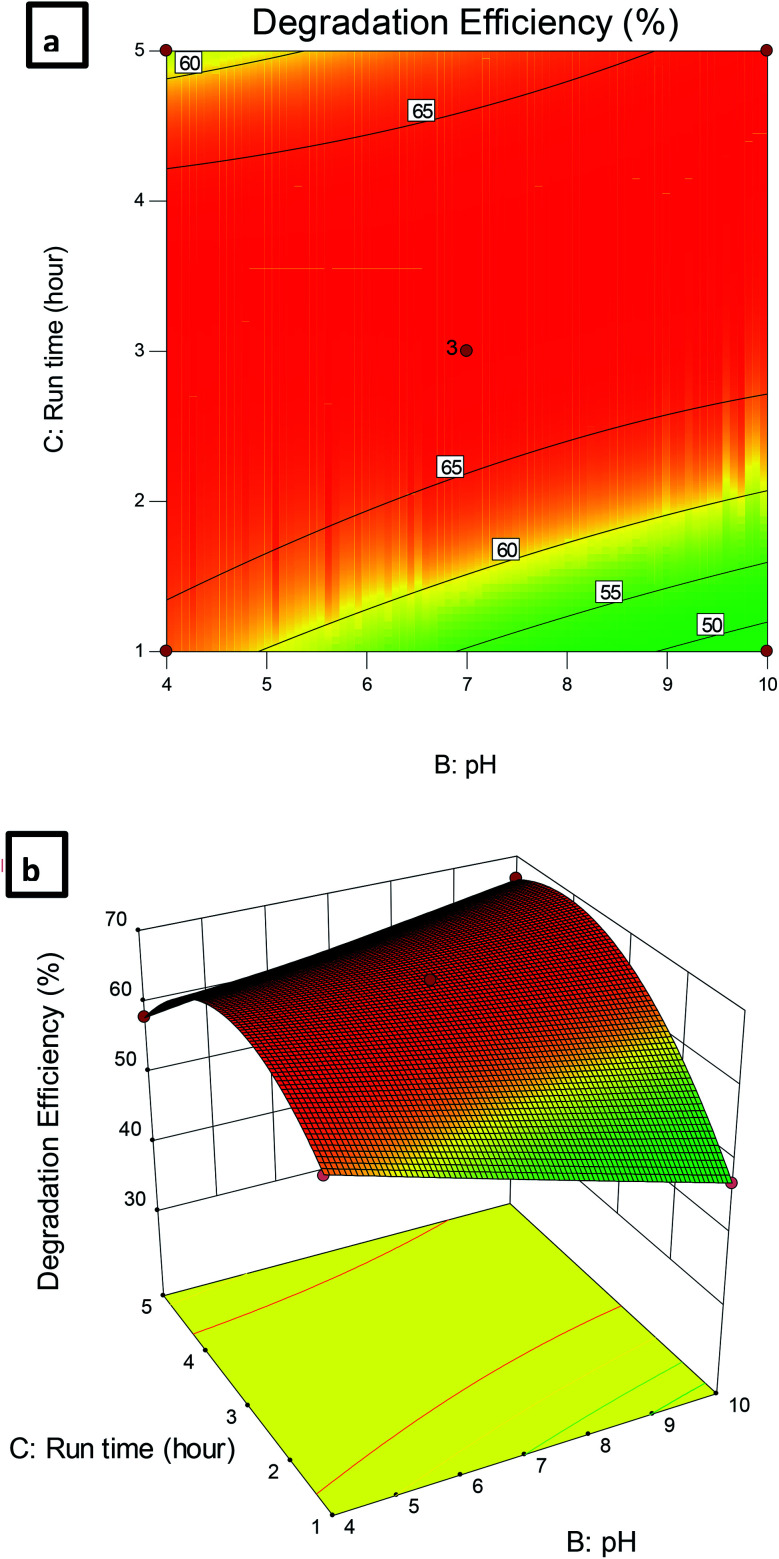
(a) Contour plot and (b) 3D plot showing the effect of pH and run time on CBZ degradation efficiency.

Fig. 10 shows the dependence of CBZ degradation on pH and run time. It can be observed that the efficiency of CBZ degradation was maximal at pH 4 and decreased with the increase of pH at all-time intervals. In addition, the increase in the time factor enhanced the CBZ remediation process up to a certain limit where no further increase in CBZ degradation was observed at all the studied pHs. Thus, based on the obtained results 0.5 g L^−1^ Pec/CS/ZnO nanocomposite, pH 4 and 3 hour run time were selected as the optimum conditions required for the remediation of CBZ in aqueous solutions with CBZ concentrations up to 10 mg L^−1^.

#### 3.3.3. Optimization of CBZ remediation process

Optimization procedure was implemented while applying a desirability approach to determine the suitable settings of the studied variables to achieve the maximum degradation of CBZ. A confirmation experiment was carried out under optimum conditions. As shown in Table 7, a value of 69.5% efficiency of CBZ degradation was obtained from the performed experiment. Such result comes in good agreement with the predicted value 69.85% confirming the validity of the proposed quadratic model to simulate the remediation of CBZ *via* a synergistic adsorption/photocatalytic technique.

The optimized CBZ degradation with observed and predicted response values^a^

**Table:** 

Independent variable	Optimized level
*X*_1_: Pec/CS/ZnO amount (g L^−1^)	0.5
*X*_2_: pH	4
*X*_3_: run time (hour)	3
Over all desirability	1.000

a *p* > 0.05.

**Table:** 

Dependent variables	Expected	Observed
*Y*: CBZ degradation efficiency (%)	69.85%	69.5%

### 3.4. Mechanism of CBZ remediation

**Fig. 11:**
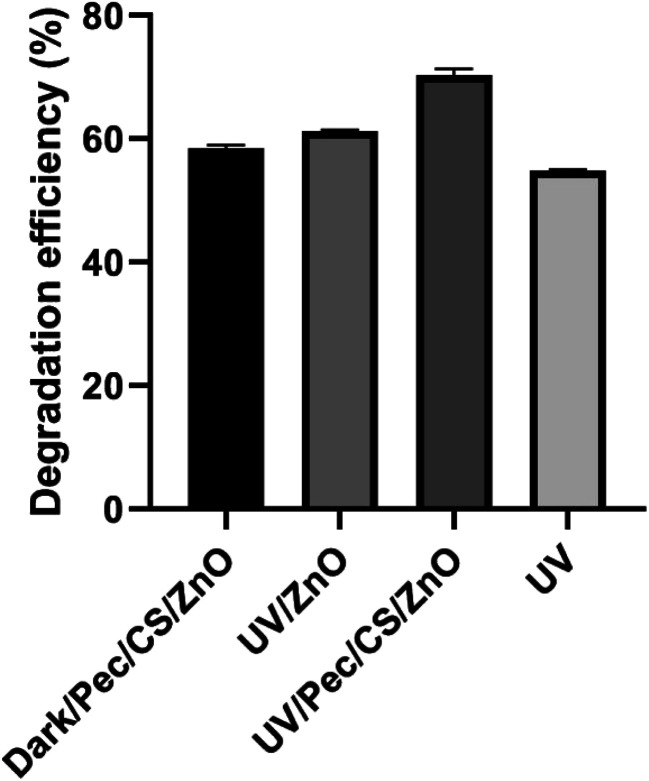
Suggested mechanism of adsorption/photocatalytic process.

For complete understanding of the mechanism of CBZ remediation, a series of reference experiments was carried out under the optimum conditions obtained by BBD using dark/Pec/CS/ZnO, UV, UV/ZnO and UV/Pec/CS/ZnO. The efficiency of CBZ degradation under these conditions is illustrated in Fig. 11. The adsorption experiment was performed in the dark in the presence of 0.5 g L^−1^ Pec/CS/ZnO nanocomposite and 10 mg L^−1^ CBZ at pH 4.0. After 3 hours, the degradation efficiency with respect to CBZ adsorption on the nanocomposite was found to be 59%. The UV-induced photocatalytic experiments were performed in the presence of 0.5 g L^−1^ ZnO nanoparticles and 0 and 0.5 g L^−1^ Pec/CS/ZnO nanocomposite at CBZ concentration of 10 mg L^−1^ for 3 hours in direct sunlight. As shown in Fig. 11, the degradation efficiency of UV, UV/ZnO and UV/Pec/CS/ZnO was 54.8, 61 and 69.5% respectively. Based on the obtained results, it can be concluded that the efficiency of CBZ degradation is enhanced in the presence of Pec/CS/ZnO nanocomposite and is based on a synergistic effect of adsorption and photocatalysis processes.

Technically, the synergistic adsorption/photocatalytic process involved the adsorption of CBZ on Pec/CS/ZnO nanocomposite and then the photo-degradation of CBZ molecules occurred. Upon illumination of ZnO in Pec/CS/ZnO nanocomposite by direct sunlight, photons were absorbed and electron–hole (e^−^–h^+^) pairs and free radicals were created.^39^ Those free radicals disrupted the conjugation in the adsorbed and free CBZ molecules in solution and degraded them.^26^ The by-products created from degradation process were then exposed for further adsorption and photo-degradation on the surface of Pec/CS/ZnO nanocomposite.^26^

The proposed mechanism for synergistic adsorption/photocatalysis degradation of CBZ is as follows:Pec/CS/ZnO + CBZ → Pec/CS/ZnO–CBZ adsorbed (in sunlight)Pec/CS/ZnO (h^+^)–CBZ adsorbed + H_2_O → Pec/CS/ZnO (OH*)–CBZ + H^+^Pec/CS/ZnO–CBZ adsorbed + OH^−^ → Pec/CS/ZnO (OH*)–CBZPec/CS/ZnO (e^−^)–CBZ adsorbed + O_2_ → Pec/CS/ZnO (O_2_^−^*)–CBZO_2_^−^* or OH* + CBZ–Pec/CS/ZnO → intermediate product → degraded product + free Pec/CS/ZnO for reuse

The kinetics of CBZ degradation using Pec/CS/ZnO nanocomposite presented a linear plot as shown in Fig. S2† and was well fitted to pseudo first order equation. The value of rate constant for CBZ degradation was 0.00737 min^−1^ as calculated from the slope of the plot and the regression coefficient (*R*^2^) was 0.9992. The *t*_1/2_ was calculated to be 94.02 min. Such kinetics of degradation is fast and suitable for wastewater treatment as compared to other reports (Table 8). Although our study showed higher *t*_1/2_ than others, it has to be taken into consideration that in the current study CBZ degradation was done on high initial concentration of CBZ (10 mg L^−1^) and sunlight was used as the source of UV light.

**Table:** Photodegradation kinetics of CBZ reported in the literature

Condition	Initial [CBZ] concentration	*K* (Kinetics)	*t*_1/2_	Ref.
UV/TiO_2_	10 mg L^−1^	0.017 min^−1^	40 min	40
UV/H_2_O_2_	42 μM	6.42 × 10^−4^ s^−1^	17.6 min	41
UV/whey–TiO_2_ NPs	0.295 μg L^−1^	0.92 per hour	—	22
UV/whey–ZnO NPs	0.295 μg L^−1^	0.84 per hour	—	22
UV/MWCNTs–TiO_2_	8 mg L^−1^	834 × 10^−4^ min^−1^	8.3 min	19
NUV-Vis/MWCNTs–TiO_2_	8 mg L^−1^	45 × 10^−4^ min^−1^	>60 min	19
UV/TiO_2_	43 mg L^−1^	0.0079 min^−1^	—	42
Sunlight/Pec/CS/ZnO	10 mg L^−1^	0.0073 min^−1^	94 min	This study

### 3.5. Reusability of Pec/CS/ZnO nanocomposite

**Fig. 12:**
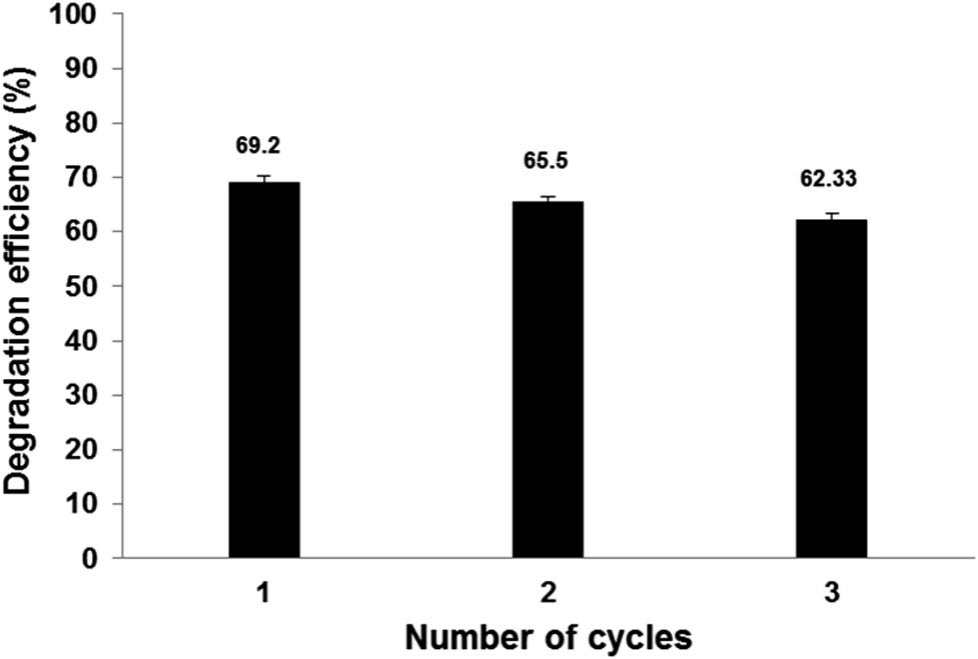
The reusability of Pec/CS/ZnO nanocomposite for CBZ degradation and removal (initial CBZ concentration: 10 mg L^−1^, pH = 4 fenitrothion and nanocomposite amount = 0.5 g L^−1^).

As observed in Fig. 12, Pec/CS/ZnO nanocomposite shows a low degradation profile. The loss of degradation efficiency between the first and the second use is negligible, although it increases in the third use most likely due to the loss of the photocatalyst ions from the composite matrix.^41^ Nevertheless, after three hours of treatment, the degradation efficiency of CBZ was slightly decreased after the third reuse which proves that Pec/CS/ZnO nanocomposite has potential stability and reusability for CBZ degradation and removal.

### 3.6. Application on synthetic wastewater samples

**Fig. 13:**
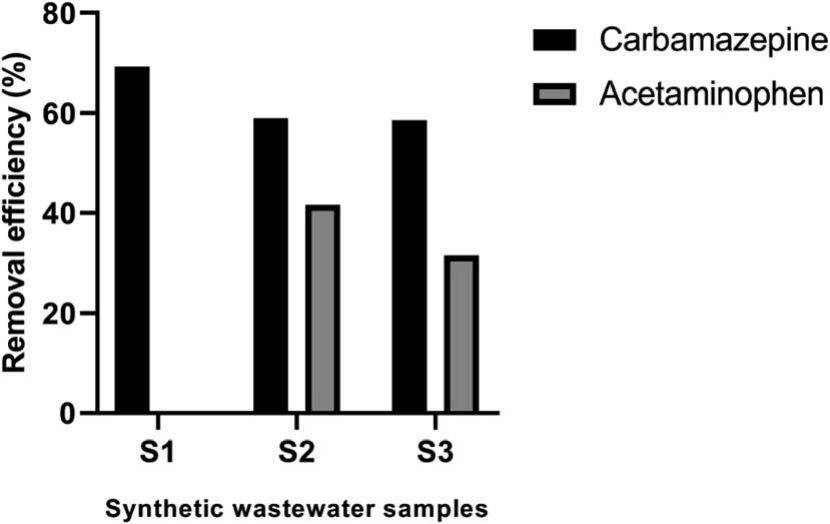
Efficiency of CBZ degradation by Pec/CS/ZnO nanocomposite in synthetic wastewater samples.

The efficiency of Pec/CS/ZnO nanocomposite for CBZ remediation in the presence of other contaminants was tested as a trial for large scale wastewater treatments. Acetaminophen was selected as model pharmaceutical contaminant. Samples were prepared by spiking distilled water with 10 mg L^−1^ CBZ and different concentration of acetaminophen ranging between 0 and 15 mg L^−1^. As shown in Fig. 13, Pec/CS/ZnO nanocomposite showed successful degradation of CBZ with an efficiency ranging between 58.57–69.30% at different levels of acetaminophen. Noticeably, the increase in acetaminophen concentration from 0 to 15 mg L^−1^ led to a slight decrease in the degradation efficiency of CBZ up to 10 mg L^−1^ acetaminophen concentration then there was stabilization in CBZ degradation with further increase in acetaminophen concentration. Such behavior could be due to the fact that acetaminophen molecules (p*K*_a_ = 9.38) might have competed with CBZ for the active sites on Pec/CS/ZnO nanocomposite thus decreasing the portion of CBZ adsorbed on the nanocomposite surface. Hence the photocatalytic degradation of CBZ was decreased. Nevertheless, the steadiness in CBZ degradation despite the addition of acetaminophen at concentrations higher than 10 mg L^−1^ could be attributed to the higher adsorption capacity of Pec/CS/ZnO nanocomposite to CBZ than to acetaminophen. Thus the competition between the two molecules on the nanocomposite active sites had an extent where any additional molecules of acetaminophen were not adsorbed and left free in the solution leading to unaffected CBZ degradation efficiency.

## 4. Conclusion

The performance of Pec/CS/ZnO nanocomposite has been evaluated for a synergistic adsorption/photocatalytic degradation of CBZ. The characterization study showed that Pec/CS/ZnO nanocomposite was an excellent choice for CBZ remediation due to its porous surface, different functionalities and high surface area. The employment of BBD to optimize the operating conditions for CBZ remediation using Pec/CS/ZnO nanocomposite was very successful. All the investigated factors showed a significant influence on the studied response. The quadratic model developed for CBZ degradation was significant with very low *p*-values (*p* < 0.0001). The optimum conditions obtained by the model included 0.5 g L^−1^ of Pec/CS/ZnO nanocomposite, pH of 4 and run time of 3 hours. The predicted values for the efficiency of CBZ degradation (%) created by the model were in good agreement with the experimental results with desirability of 100%, and 69.5% degradation efficiency. The presence of other pharmaceutical contaminants like acetaminophen in wastewater samples did not have a significant effect on the remediation of CBZ using Pec/CS/ZnO nanocomposite. The obtained degradation efficiency was almost constant at different concentrations of acetaminophen.

Moreover, the study clearly showed that Pec/CS/ZnO nanocomposite employed in a synergistic adsorption/photocatalytic degradation of CBZ was one of the appropriate methods for the remediation of serious pharmaceutical contaminants as CBZ. Further understanding and elaboration of the kinetics, mechanisms, intermediates produced during the degradation process will be performed in future work.

## Conflicts of interest

The authors declare that there is no conflict of interest.

## Supplementary Material

RA-010-D0RA08010A-s001
