# Supplementary material for: A pectin/chitosan/zinc oxide nanocomposite for adsorption/photocatalytic remediation of carbamazepine in water samples
Source: RSC Adv. 2020 Nov 9;10(67):40697–708. doi: 10.1039/d0ra08010a (PMC9057706; doi:10.1039/d0ra08010a)
Supplement: RA-010-D0RA08010A-s001 [file RA-010-D0RA08010A-s001.pdf]

Electronic Supplementary Material (ESI) for RSC Advances.
This journal is © The Royal Society of Chemistry 2020

# "Pectin/Chitosan/Zinc Oxide nanocomposite for adsorption/photocatalytic remediation of carbamazepine in water samples"

## Electronic Supporting Information (ESI)

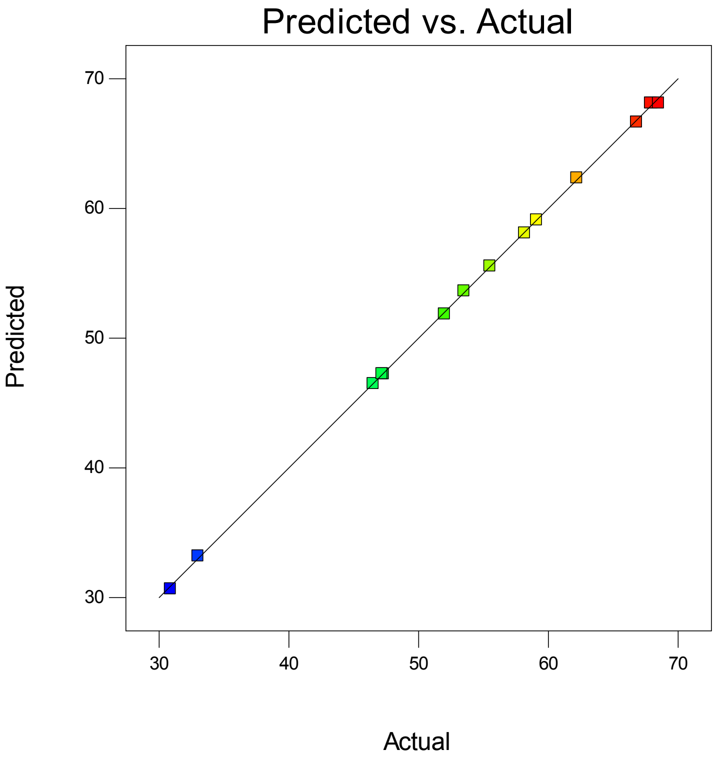


**Figure S1: Plot of predicted values versus actual values for CBZ degradation using Pec/CS/ZnO nanocomposite**

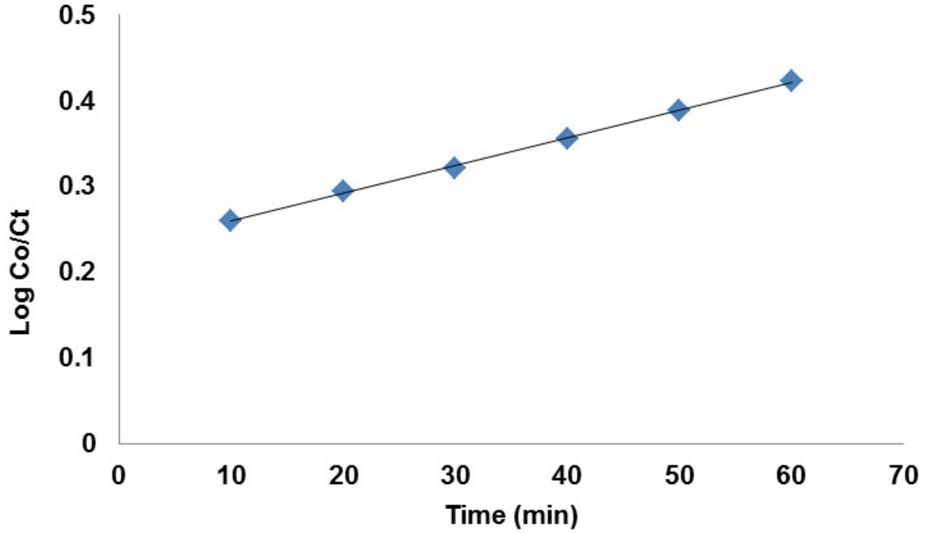


**Figure S2: Fitting of kinetics data to Pseudo first order equation, Pec/CS/ZnO amount: 0.5 g/L, CBZ conc.: 10 mg/L, temperature: 25 °C, pH: 4.0**
